# Cryoablation for atrial fibrillation: biophysics and a contemporary step-by-step approach

**DOI:** 10.1016/j.ipej.2026.02.002

**Published:** 2026-02-04

**Authors:** Jyothi Vijay, Narayanan Namboodiri

**Affiliations:** Division of Cardiac Electrophysiology, Sree Chitra Tirunal Institute of Medical Sciences and Technology, Kerala, India

**Keywords:** Atrial fibrillation, Cryoablation, Cryoballoon, Pulmonary vein isolation

## Abstract

Cryoablation has become the standard of care for treating atrial fibrillation (AF). Over the past two decades, significant advances have been made in this field. This review discusses the evidence-based practical aspects and recent advances in cryoablation for AF, with a focus on biophysics, procedural techniques, and evidence for cryoballoon-based pulmonary vein isolation (CBA).

## 1 Introduction

Atrial fibrillation (AF) is a widely prevalent cardiac arrhythmia. Despite its progressive nature and myriad complications, especially the stroke risk, treatment of this condition remains far from perfect and is evolving rapidly. Contemporary treatment of AF focuses on catheter ablation of triggering pulmonary vein potentials and antiarrhythmic therapy. Cryoballoon-based ablation (CBA) has established itself as a safe and effective technology for isolating the triggering foci within the pulmonary veins. Pulmonary vein isolation can restore and maintain sinus rhythm, reduce heart failure admissions and stroke risk, and improve the quality of life [1,2].

## 2 Biophysics of cryoablation

### 2.1 Mechanism of cryogenic injury

Current cryoablation balloons work by ultrafine injection of highly pressurized liquefied nitrous oxide from a cylinder kept at the console into a low-pressure zone in the balloon, producing a cooling effect. It works on the principle of the Joule-Thomson effect (expanding gas in a region of low pressure causes cooling) [3]. The boiling point of nitrous oxide, N2O, is −88.48 °C at standard atmospheric pressure. Joule Thomson effect requires the following conditions to be met. Firstly, gas moves from a high-pressure area to a low-pressure area through a small opening. Secondly, it requires an insulated system to prevent heat loss to the surroundings through adiabatic expansion [4]. After absorbing heat from tissue at contact, the refrigerant returns to the console and is disposed of through the hospital gas scavenging system. Radiofrequency ablation works by generating resistive heating at the catheter-tissue interface. In contrast, cryoablation works through cooling and crystal formation.

**Fig. 1:**
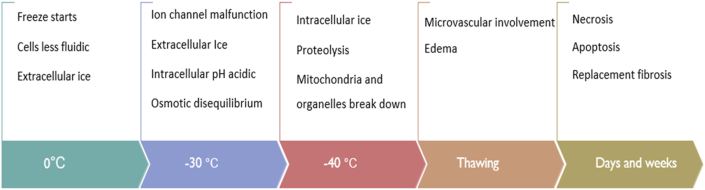
The sequence of thermal effects in cryoablation [3,5].

Cryogenic injury occurs in three phases. An initial phase characterized by thermal injury by cooling (freeze-thaw phase), an intermediate phase with secondary changes in the centre (inflammatory phase), and a final phase of apoptotic cell death in margins and replacement fibrosis [3,5]. Tissue damage occurs in phases, with an immediate, direct thermal injury followed by cellular damage, apoptosis, and, eventually, a late wave of replacement fibrosis [6]. Ice crystal formation inside and outside the cell is a critical event that triggers a cascade of events leading to a cryoablation lesion. The affected tissue becomes less fluidic initially (Fig. 1). The critical temperature for irreversible injury is −30 °C. The slower the temperature recovery, the more tissue damage occurs [7]. Also, repeating the freeze-thaw- freeze cycle increases the lesion depth, especially into the periphery of the lesion. Ice recrystallization and osmotic damage are increased by longer thaw times [8].

### 2.2 Determinants of the cryo-effect

Determinants of volume of tissue frozen after a single application of cryoablation include convective warming, catheter tissue contact, electrode size and orientation, electrode contact pressure, catheter refrigerant flow rate, and nature of the tissue. A larger lesion can be produced with an 8 mm electrode than with a 4 mm electrode [9]. In the study by Wood et al., [9] tissue temperature was measured at various depths, and horizontal orientation resulted in lower temperatures than vertical orientation. Longer delivery produces a larger lesion, and vice versa.

### 2.3 The cryolesion

Reports of histopathological correlates of cryoablation lesions in the human heart are rare. CBA lesions showed fibrous granulation tissue with myocardial disruption and transmural lesions, whereas the gross pathologic appearance was not obvious, unlike RFA [10]. Cryoablation results in a lower incidence of thromboembolic complications and less thrombus volume than radiofrequency ablation, possibly due to less tissue distortion [11]. Cryotherapy lesions preserve tissue ultrastructural integrity, and the absence of collagen damage is proposed as an important factor. Collagen and fibroblasts resist cryothermal injury [12]. LIPV has been shown to exhibit the greatest circumferential late gadolinium enhancement on follow-up cardiac magnetic resonance imaging. Patients without recurrence are noted to have more completely circumferentially treated veins [13]. In the IVUS‐Cryo Study, an intravascular ultrasound (IVUS) study conducted before and after PVI using CBA, the effects are not limited to the PV ostium. Key IVUS changes noted in this study are pulmonary vein oedema with the formation of dissection-like spaces within [14].

Biophysical factors favouring CBA over RF are given in the table (Table 1) [12,15].

**Table 1:** Biophysical factors favouring cryoablation over RF.

Biophysical factors favouring cryoablation over RF
• Sharply demarcated lesions
• Preserved vascular ultrastructural integrity
• Cryo-adherence (help catheter stability)
• Lesser thrombogenicity
• Stable tissue-catheter contact
• Possibility of single-shot freeze

## 3 Comparison of cryoablation versus RF for PVI

After the demonstration of the importance of pulmonary vein triggering foci in AF by the seminal work of Haïssaguerre et al., [16] PVI has been the cornerstone of AF care. The Cryo-AF Global Registry was a large, multicentre, prospective registry evaluating cryoablation for AF. Serious procedural and device-related adverse event rates were 1.5% and 3.4%, respectively. At 1-year follow-up, 90% of patients with paroxysmal AF were free of AF [1]. Major studies that have shown cryoablation to be non-inferior to RF PVI were the “Fire and Ice” trial, German ablation registry, and the “Freeze” cohort study. In the “Fire and Ice” trial, after 1.5 years of follow-up, CBA was found to be non-inferior to RFA for drug-refractory paroxysmal AF [17]. In patients who underwent re-ablation in the “Fire and Ice” trial, CBA-treated patients had fewer PV reconnections, with similar acute success rates [18]. In the Freeze prospective cohort study, CBA was shown to be quicker and associated with a lower recurrence risk than RF in paroxysmal AF [19].

In the NO-PERSAF study, CBA for persistent AF had a shorter procedural duration, similar rates of freedom from AF, and a lower incidence of atrial flutter compared with the RF group at 12 months, and these results were sustained at 3-year follow-up [20]. In their large meta-analysis of 8668 patients, Cardoso et al., [21] concluded that freedom from AF is similar in the CBA group and RF(with or without contact force technology). Gap distribution in redo procedures also differs between CBA and RFA. Redo ablations after CBA were quicker than after RFA. Gaps were more common in RSPV after initial RF [22].

## 4 Preprocedural planning

A preprocedural transthoracic echocardiogram is performed to assess left ventricular function, LA size, the septum, and the pulmonary veins, and to exclude an intracardiac clot. A transesophageal echocardiogram can assess the interatrial septum for transseptal catheterization and exclude a left atrial appendage clot without radiation risk.

A preoperative CT scan, though not mandatory, can help reveal abnormal pulmonary vein anatomy, such as an additional pulmonary vein or a common pulmonary vein (most commonly on the left side). Cardiac CT also helps to exclude a thrombus in the left atrial appendage and to rule out a septal aneurysm or patent foramen ovale. A dedicated CT protocol can be helpful for image fusion with real-time 3D images if electroanatomic mapping is used during cryoablation. The cranial and caudal limits can be defined as the base of the LA (up to the left pulmonary vein insertion) and the mitral annulus (coronary sinus as the marker for the first slice), resulting in many thin slices (around 25) depending on the atrial size. The space demarcated by the posterior LA myocardium and the manually traced pericardium can be used for quantification.

## 5 The cryoablation procedure

The procedure can be done with sedation and a laryngeal mask airway or general anesthesia. Muscle relaxants that paralyze the diaphragm are usually avoided.

### 5.1 Access and catheters

Vascular access includes the bilateral femoral veins. Right radial artery access can be used for arterial BP monitoring. The 6-French venous access is used to place a quadripolar catheter at the Hisian location. The same catheter is advanced into the superior vena cava (SVC) at the SVC-subclavian junction to capture the phrenic nerve and assess its integrity during right-sided PVI. Left-sided venous access can be used for an intracardiac echocardiography (ICE) catheter. If available, the right jugular venous access can be used to position a decapolar catheter in the coronary sinus (CS). This can help with CS pacing to better discern PV potentials, perform a basal electrophysiologic (EP) study, induce, and test. It's imperative to perform a basic electrophysiological study to rule out accessory pathway-mediated supraventricular tachycardia or focal atrial tachycardia arising outside the PV as a driver of AF. Full anticoagulation is achieved with intravenous heparin, maintaining a target activated clotting time of 350 s.

### 5.2 Left atrial access

Transseptal catheterization through the lower anterior fossa ovalis can be achieved under fluoroscopic or echocardiographic guidance. The cryoablation sheath has a relatively abrupt step at the sheath dilator assembly, which can make LA entry difficult at times. Prior septal dilation with smaller serial dilators can resolve this issue. In challenging cases, a quadripolar catheter placed in the left atrium (LA) can facilitate parallel sliding entry of the steerable sheath. While advancing the sheath into the LA, the quadripolar catheter is pulled back, similar to putting on a shoe with a shoehorn [23]. Alternatively, inserting a stiff wire into the right superior pulmonary vein can sometimes facilitate entry [24]. Once the sheath is placed into the LA across the septum, it needs to be de-aired and flushed with continuous, slow saline infusion.

### 5.3 Preparing the cryoballoon system

While preparing the CB, avoid wetting the proximal end of the catheter, especially where electrical and gas coaxial cable connections are made, by placing a dry gauze between the electrical and gas ports and the balloon's proximal hub. It is preferable to connect a 3-way manifold via a Y-connector (a Tuohy-Borst adapter) to the balloon catheter's side port. Attach a pressure line to one of the manifold's ports. The other port of the manifold can be used for contrast, and the third for saline. Then, introduce the circular wire mapping catheter using the introducer by opening the Y connector. Flush distally and backward with a 20 cc syringe through the Y connector, ensuring the system is leak-free and air-free. Then the balloon is introduced with the protective sleeve in place.

### 5.4 Step-by-step isolation of the pulmonary vein

1. Through the steerable sheath (FlexCath Advance, Medtronic), the 28 mm cryoballoon (Arctic Front Advance, Medtronic) is introduced over the circular wire mapping catheter (Achieve Advance, Medtronic). The CB catheter assembly can be directly advanced into the PV and the wire-mapping catheter should always be ahead of the stiff balloon tip. The Flexcath sheath can be directed towards the pulmonary vein. The circular mapping catheter can be used to map PV potentials. There are two white marker bands on the balloon catheter. The first one indicates that the balloon's distal tip is at the tip of the FlexCath sheath. The second one, once it reaches the hub of the steerable sheath, the entire balloon is out into LA.
2. Once the mapping catheter is within the PV, a slight pull on the wire can help track the balloon. It's advisable to avoid inflating within the pulmonary veins. Balloon occlusion can be confirmed with a contrast venogram, by documenting the occlusion pressure tracing, or with ICE. During CBA, a delay and eventual disappearance of PV potential can be observed. The catheter can be slightly pulled back to regain the circular loop for recording potentials after balloon inflation prior to the freeze.
3. Segmental occlusion can be performed using balloon deflection. Balloon deflection can help to ensure apposition for segmental isolation. Using the inferior branch of the inferior veins for entry provides better occlusion. In difficult cases, especially with the right inferior pulmonary vein, once the sheath is directed toward the target PV, a *“fishing/probing manoeuvre”* can be performed with the mapping catheter to enter the target PV. A “*Hockey stick manoeuvre*” can be used to enter inferior veins, especially when the balloon is not coaxial, or the location of septal puncture is not favourable (video 1). A LAO view can be used as a default view for left PVI and an RAO view for right-sided PVI. Pulmonary vein entry can be facilitated by a 3D electroanatomic map if available, though it is not essential.
4. The *proximal seal technique* helps to position the balloon in the antrum and reduce collateral damage. After the venogram, the balloon catheter is gently withdrawn to allow a small amount of contrast leak, which prevents freezing within the pulmonary vein. Then the balloon is minimally pushed to maintain contact at the antrum.
5. For esophageal temperature monitoring, the esophageal temperature sensor is positioned fluoroscopically as close as possible to the left-sided PV being treated. A format for the CBA case observation chart is given below (Table 2).

**Table 2:** A format for Cryoablation case observation chart.

Serial number of freezes	Vein	Nadir temp	Esophageal Temperature at start	Esophageal Temperature at stop	Time to −30 °C	Time to isolation	Time to thaw
	LSPV						
	LIPV						
	RIPV						
	RSPV						
LA dwell time in minutes							
Contrast in ml							
Fluoroscopy time

Supplementary data related to this article can be found online at https://doi.org/10.1016/j.ipej.2026.02.002

The following are the Supplementary data related to this article:

Video 1: Hockey stick maneuver for cryoballoon occlusion.Multiemedia component 2

The balloon should be rewrapped with an inflate-balloon extend-deflate manoeuvre every time for a smooth re-entry into the sheath. Exit and entry blocks can be checked after the procedure in all veins in sinus rhythm and during pacing. The femoral access site can be secured with a figure-of-eight suture after sheath removal.

## 6 Safety considerations

Complications during CBA, though rare, include pericardial effusion, stroke risk, and collateral damage to the right phrenic nerve, esophagus, and left main bronchus.

### 6.1 Protecting the phrenic nerve

Phenic nerve injury is a known complication with a reported incidence of 4.2% during CBA in large studies like the YETI registry [25]. Fortunately, 97% of cases recovered at one year of follow-up. *YETI score*, based on factors such as age, use of the bonus freeze protocol, nadir temperature, a 30% or greater reduction in CMAP amplitude, and active deflation by double stop, can predict one-year recovery after CBA [25]. Pulmonary vein cryoablation: the usual sequence is the LSPV, then proceed clockwise to end in the RSPV. This sequence is based on the principle that PVI is almost complete even if phrenic nerve dysfunction occurs during RSPV cryoablation. RSPV CBA carries a higher risk of phrenic nerve injury than inferior PV cryoablation (5.5% versus 0.7% in a large study) [26].

**Fig. 2:**
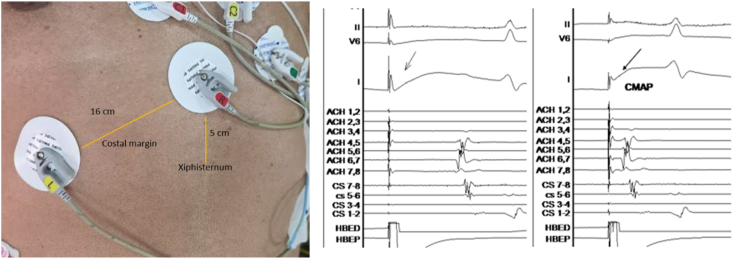
Placement of surface ECG electrodes for recording of diaphragmatic compound muscle action potential(*left*). CMAP during the start of cryoablation(*middle)*and later showing a reduction in CMAP during the development of phrenic injury(*right*).

Diaphragmatic neurological integrity can be monitored through recording of the diaphragmatic compound muscle action potential CMAP, fluoroscopy, fetal heart monitor, ICE, and by direct palpation over the abdomen. A quadripolar catheter with a curve (e.g., CRD2 Catheter, Abbott) can be used to achieve stable phrenic capture at the SVC or the SVC-subclavian vein junction. Another method to record CMAP is to place a quadripolar catheter in the inferior vena cava across the diaphragm. Usually, the PN capture threshold is checked, and pacing is started at twice the threshold at a cycle length of 1000-1200 ms. Phrenic pacing typically begins around 15 s after freezing starts, allowing the temperature to drop below zero. Diaphragmatic CMAP can be recorded using surface ECG electrodes, with one electrode positioned 5 cm above the xiphoid process and the other 16 cm along the costal margin. If a 30% decrease in CMAP amplitude occurs, additional freezing is stopped by actively deflating the balloon with a double-stop method (Fig. 2). A CMAP amplitude cutoff of 0.2 mV during emergent balloon deflation predicts the likelihood of phrenic recovery within 1 day [26].

The “NeedMap” is an alternative method for monitoring diaphragmatic CMAP by placing ECG electrodes at the anterior axillary line, with better sensitivity and specificity for PNI [27]. Female sex and low BMI may be predictors for the development of phrenic nerve palsy. Most cases of phrenic nerve palsy resolve in a few months [28].

### 6.2 Esophagus

It's good practice to stop freezing once esophageal temperature falls below 25 °C. Often, temperatures drop several degrees even after the freeze ends, before recovery. Adequate temperature recovery should be obtained prior to the next freeze. The risk of atrioesophageal fistula is 1 in 25000 patients and is associated with a delayed presentation compared with RF [29].

## 7 Tailoring the cryoablation dosing

A single 3-min ablation strategy may be equally effective, and a bonus freeze may not be required in most cases if acute isolation is achieved quickly [30]. A protocol for cryodosing based on TTI can individualize therapy and reduce late PV reconnections and atypical atrial tachycardias on follow-up. A common rule of thumb is that if TTI is under 60 s, total therapy for 180 s is given for right-sided veins. If TTI exceeded 60 s, the total therapy time is 240 s. For left-sided veins, a total of 240 s of CBA is given. If no isolation is observed within 90 s, freezing can be stopped, and the balloon is repositioned. The left inferior pulmonary vein may not achieve lower nadir temperatures than other pulmonary veins, possibly due to its proximity to the mitral valve, but fortunately, reconnection is less common in LIPV [31]. The inferior part of the RIPV has a predilection for PV reconnection and is often the most frequently reconnected PV during redo procedures. But bonus freezes don't add to the efficacy of PVI [32]. In the Spanish registry, time to reach −30 °C has been shown to predict successful PVI. Moreover, a 10-s increment in time to reach −30 °C reduces the odds of successful PVI by 41% [33]. A comparison of various dosing strategies is compiled in table (Table 3).

**Table 3:** Various studies comparing different cryodosing strategies.

Study	Approach	Effect	Remarks
Aryana et al. [34]	Time to isolation of PV guided If TTI <60 s = TTI+ 2 min single shot If TTI >60 s = a bonus 2min dose added TTI not clear = 3 + 2 min	Higher PVI durability Less atypical atrial tachycardias	Non-randomized study
Ciconte et al. [30]	Three-minute versus 4-min Freeze Strategy	Equal efficacy for both approaches	Bonus freeze unnecessary
Chen et al. [35]	TTI guided 240 s vs 180 s	TTI-guided 240-s freeze duration has a higher rate of durable PVI	ICE Re-Map Study
Nakano et al. [36]	150 s if ≤ −40 °C within 40 s 120 s if the ≤ −50 °C within 60 s Other patients 60 s	Both groups have similar AF free survival at one year. Shorter procedure time and RF touch-up in the tailored dose group	Non-inferiority study
Miyazaki et al. [37]	Different criteria for each PV	**LSPV**: faster freeze to −40 °C **LIPV**: faster freeze to −40 °C **RSPV**: lower nadir temperature, slower thawing speed, and smaller PV size **RIPV**: slower thawing speed	Best freeze criteria per PV

## 8 Special situations

### 8.1 Anatomical challenges

Left common PV (LCPV) is a common anatomical variation. In the left common PVI, segmental isolation can be achieved by advancing the mapping catheter into each PV branch to guide balloon occlusion. A preprocedural CT can help predict the alignment of the LCPV and the inferior branch, which might contribute to a difficult occlusion. A meta-analysis of 5 published studies found that the presence of LCPV does not affect the long-term outcome or Safety of CBA with a 28 mm balloon [38]. Using a size-adjustable CB (28 mm or 31 mm) may be better than a fixed 28 mm balloon in terms of success rate and freezing time [39].

If the inferior PV has a gap at its lower part, the *“pull-down maneuver*” can be helpful (video 2). After 60 s of freeze, the CBA is gently pulled down to increase tissue contact at the inferior PV. This should be done very gently as there is a risk of tissue injury [40]. An interatrial septal aneurysm and a thick septum can make transseptal access difficult. RF. Esophageal abnormalities, such as stricture, and vascular anomalies, such as Kommerel's diverticulum, if present, can affect the placement of the esophageal temperature probe (Table 5).

Supplementary data related to this article can be found online at https://doi.org/10.1016/j.ipej.2026.02.002

The following are the Supplementary data related to this article:

Video 2: *Pull-down maneuver* for sealing an inferior gap in the inferior pulmonary veins.Multiemedia component 4

### 8.2 Large LA

Patients with paroxysmal AF and a markedly enlarged LA have outcomes comparable to those with an LA size less than 40 mm [41]. In cases of incomplete isolation with CBA, RF touch-up can be used to complete PVI or to modify the substrate via the posterior or mitral line, as required. This is more relevant to patients with advanced disease and diseased atrial substrates.

### 8.3 Presence of atrial septal occluder

Transesophageal echocardiography-guided entry through the native septum can be considered for most patients with an adequate native septum and an atrial septal occluder device diameter less than 25 mm [42]. Difficulties in crossing the septum can be overcome by gradually dilating the septum over the guidewire. Pigtail wire (jalebi wire) can facilitate crossing a difficult-to-track septum.

## 9 Role of intracardiac echocardiography (ICE) and electro-anatomical mapping

**Fig. 3:**
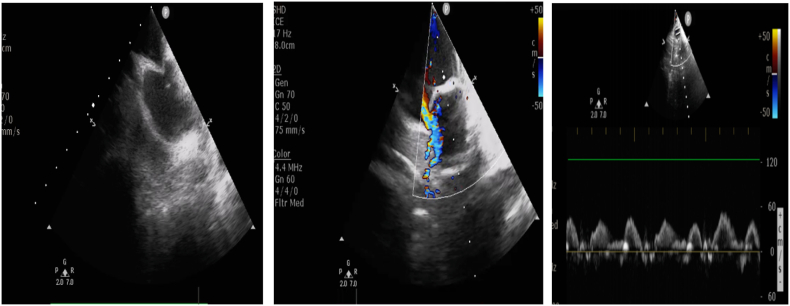
The utility of ICE in cryoablation. (*Left),* tenting of the interatrial septum by the septal puncture needle during LA entry (video 3), (*middle)*, image assessing for leak during balloon occlusion and monitoring of phrenic nerve function using pulsed wave Doppler using ICE (right).

Intracardiac echocardiography can assist transseptal catheterization and balloon occlusion (Fig. 3). Leak upon balloon occlusion in the pulmonary vein can appear with a “golf on a tee” appearance [24]. The right phrenic nerve can be directly visualized by ICE. This can also guide the pacing capture threshold, but there is no data on whether it helps reduce the incidence of phrenic nerve palsy [43]. Phrenic nerve function can be monitored using pulsed-wave Doppler with ICE (Fig. 3). The utility of ICE in CBA is summarized in the following table (Table 4).

**Table 4:** Utility of intracardiac echocardiography in cryoablation.

Utility of intracardiac echocardiography in cryoablation
• Visualization of the interatrial septum
• Excluding LA or LA appendage thrombus
• Confirming LA entry
• Assessing the “seal” of the balloon on PV occlusion
• Monitoring for phrenic nerve injury
• Identifying catheter-related thrombus
• Monitoring for pericardial effusion

**Table 5:** Common problems observed during CBA and troubleshooting.

Problem observed	Possible reason	Solution
Difficult entry of the sheath at the femoral site	Inadequate dilatation of access. Previous procedure scar.	Dilate the access with serial dilators. Ultrasound-guided access
CMAP of poor quality	Related to ECG position, electrodes, or body habitus	Change the electrodes to remove artefacts. Use a quadripolar catheter in the IVC for CMAP, or use alternative methods of PN monitoring
Difficult to cross the interatrial septum	Thick septum Inadequate septal dilatation	Serial dilatation of the septum. Shoehorn technique [23]. Use of a soft-tipped extra support guidewire. Engaging the wire in RSPV and tracking for better alignment
Significant contrast leak during balloon occlusion	Ovoid PV ostium Inadequate occlusion	Engagement of a different branch PV with a segmental approach
Difficult occlusion in the inferior PV	Abnormal alignment of the PV or trans septal site or poor tissue catheter contact	Try entering the inferior PV branch for better occlusion. Hockey stick manoeuvre [24]. Pull-down manoeuvre [40].
Incomplete pulmonary vein isolation	Poor balloon contact or positioning	Optimise balloon placement and confirm vein occlusion
Hypotension during freeze	Vagal or pseudo vagal response to ice crystals [46]	Usually, a transient phenomenon. Response to atropine is variable.
Balloon deflation	Hardware issue	Change the coaxial umbilical cable. If persistent, change the catheter

Supplementary data related to this article can be found online at https://doi.org/10.1016/j.ipej.2026.02.002

The following are the Supplementary data related to this article:

Video 3: Transseptal catheterization for cryoablation guided by intracardiac echocardiography. Tenting of the interatrial septum can be seen.Multiemedia component 3

**Fig. 4:**
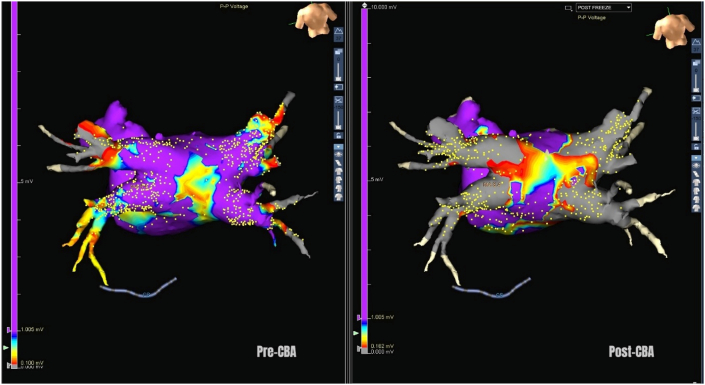
Pre- and post-images of cryoablation PVI created with electroanatomic map-CT fusion imaging.

Few studies have reported the incremental value of 3D electroanatomic mapping during CBA [44].Merging electroanatomic mapping with CT can be useful for identifying PV ostia and mapping veins (Fig. 4). This can be performed using the built-in software in the 3D electroanatomic mapping system (e.g., Ensite Verismo, Abbott, United States) to segment cardiac CT data. Nevertheless, electroanatomic mapping can show the additional area of posterior wall isolation achievable with CBA beyond PVI. This additional ablation, when combined with electroanatomic mapping, can further debulk the posterior LA beyond PVI [45]. Whether this may contribute additively to CBA efficacy is not clear.

## 10 Troubleshooting

Common practical issues encountered in the procedure, their possible reasons, and solutions are summarized (Table 5).

## 11 Future directions

### 11.1 HPSD and hybrid approach

CBA is not without limitations. When high-power short-duration ablation is compared with CBA, both approaches had comparable safety and efficacy for AF. HPSD may be superior at eliminating extrapulmonary triggers. Data on the hybrid approach for atrial fibrillation is limited. Sequential hybrid ablation, rather than surgical cryomaze alone, may result in better freedom from AF/AT. An epi-endocardial combined ablation cohort demonstrated outcomes comparable to those of the cryoballoon group and fewer antiarrhythmic prescriptions [47]. Patients with prior ablation failures and persistent AF are more likely to be candidates for this hybrid approach.

### 11.2 Ultra-low-temperature CBA

Ultra-low-temperature CBA is performed with catheters that use “near-critical” nitrogen, which has a boiling point of −196 °C, compared to nitrous oxide, which has a boiling point of −89 °C. Initial studies are encouraging, and if proven in larger trials, this may offer better results in the persistent AF population. This technology also enables cryomapping to rule out phrenic involvement [48].

### 11.3 Size-adjustable cryoballoon

**Size-adjustable** CBA offers greater versatility in selecting balloon size and a higher proportion of single-shot freeze PVI [49]. Multicenter randomized studies are in the pipeline to evaluate size-adjustable versus conventional CBA patients with paroxysmal AF. Cryoablation is more reproducible and has less inter-operator variability than RFA. This holds true with the newer size-adjustable CB as well.

### 11.4 Dual energy ablation

Ultra-low-temperature cryoablation and pulsed-field ablation (PFA) can be combined in a single catheter and shown to be feasible and to achieve good acute success in the multicenter first-in-human PARALLEL trial. This approach may be attractive because catheter contact stability and effectiveness can be maintained with dual energy [50].

## 12 Conclusion

Cryoablation offers PVI with good procedural safety and long-term efficacy, particularly in paroxysmal atrial fibrillation. With a shorter learning curve and widespread availability, it can provide relief to a significant subset of patients with AF. Cryoablation is likely to remain in the electrophysiologist's armamentarium for treating AF despite the advent of newer energy modalities.

## Disclosures

The authors have no conflicts of interest to disclose.

## Approval

All authors have seen and approved the manuscript.

## Credit author statement

All authors have equally contributed to conceptualization, review of literature, writing, and editing of the draft.

## Funding

No funding/financial support received for this work.

## Declaration of competing interest

The authors declare that they have no known competing financial interests or personal relationships that could have appeared to influence the work reported in this paper.

## List of abbreviations

AF: Atrial Fibrillation
AT: Atrial tachycardia
CB: Cryoballoon
CBA: cryoballoon ablation
CT: Computed Tomography
HPSD: High Power Short Duration
ICE: Intra Cardiac Echocardiography
IVUS: IntraVascular Ultrasound
LAO: Left Anterior Oblique
LCPV: Left Common Pulmonary Vein
LIPV: Left Inferior Pulmonary Vein
LSPV: Left Superior Pulmonary Vein
PNI: Phrenic Nerve Injury
PVI: Pulmonary Vein Isolation
RAO: Right Anterior Oblique
RCT: Randomized controlled Trial
RFA: Radio Frequency Ablation
RSPV: Right superior Pulmonary vein
RIPV: Right Inferior Pulmonary Vein
TTI: Time To Isolation
