# Supplementary material for: Cryoablation for atrial fibrillation: biophysics and a contemporary step-by-step approach
Source: Indian Pacing Electrophysiol J. 2026 Feb 4;26(1):5–12. doi: 10.1016/j.ipej.2026.02.002 (PMC12958032; doi:10.1016/j.ipej.2026.02.002)
Supplement: Multimedia component 1 [file mmc1.docx]

**Supplementary material**

**Video 1: Hockey stick maneuver for cryoballoon occlusion**

**Video 2: *Pull-down maneuver* for sealing an inferior gap in the inferior pulmonary veins**

**Video 3: Transseptal catheterization for cryoablation guided by intracardiac echocardiography. Tenting of the interatrial septum can be seen**


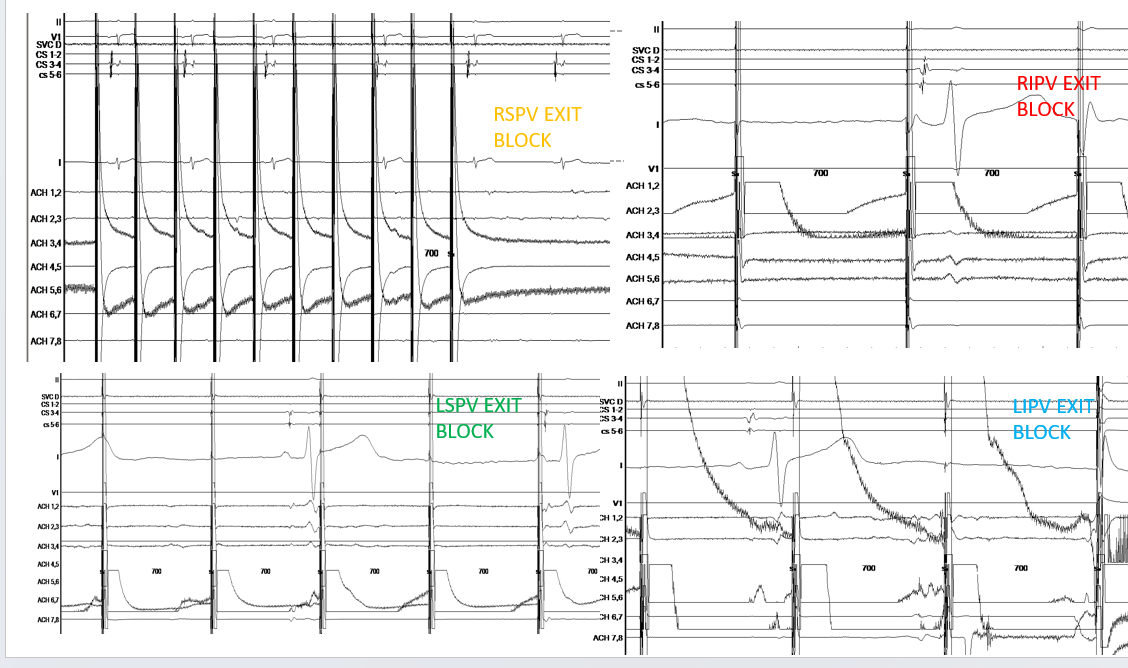


**Figure 5: Exit block can be demonstrated in all pulmonary veins after cryoablation. Pacing is performed via a catheter in the pulmonary veins. (ACH-achieve advance pacing catheter)**


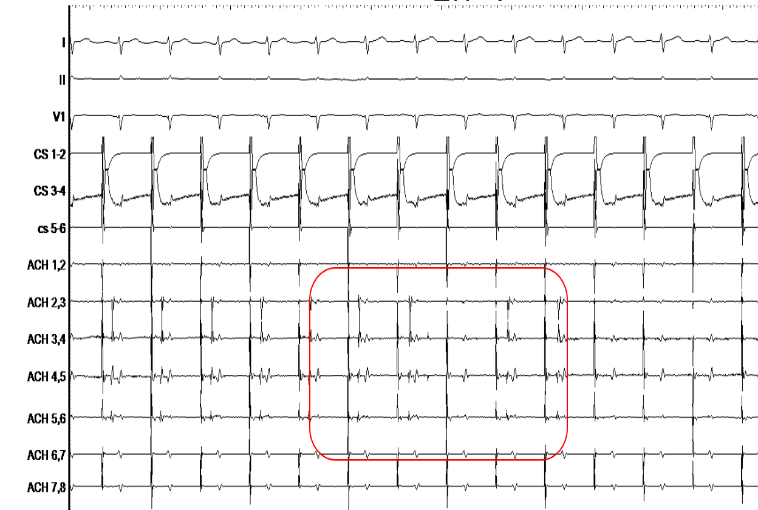


**Figure 6: Loss of pulmonary vein potential with cryoablation. Coronary sinus is being paced and recordings from achieve advance catheter(ACH) is highlighted.**


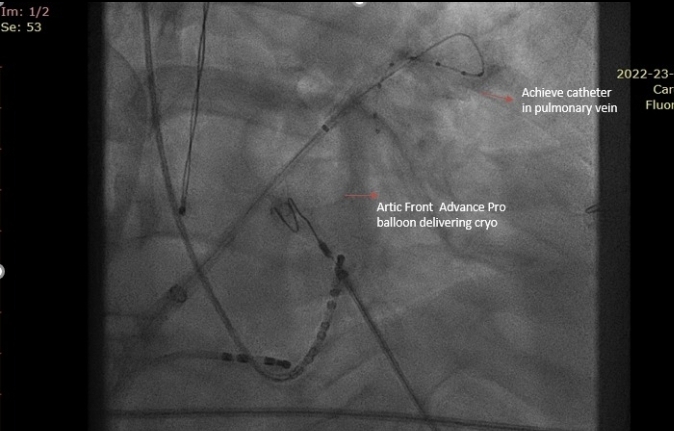


**Figure 7: Labelled fluoroscopic image of cryoablation using an artic front advance pro balloon catheter in RAO view. The esophageal temperature probe can also be seen.**


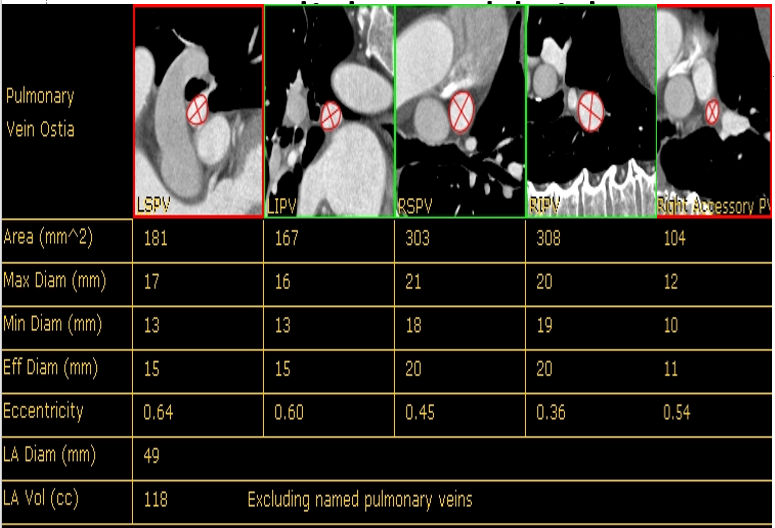


**Figure 8: An example of a reporting format for a dedicated Cryoablation CT protocol**


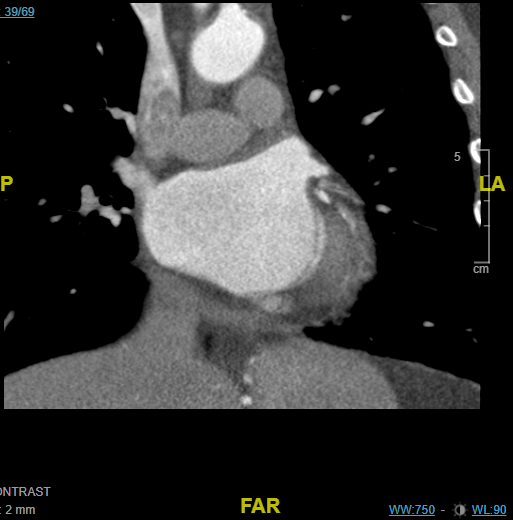

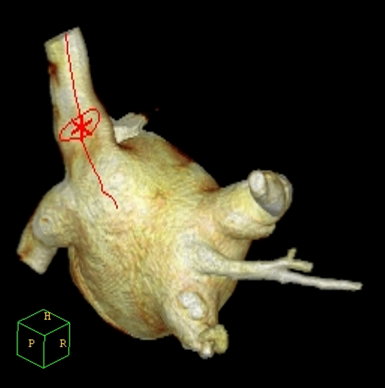


**Figure 9: Anatomical differences in pulmonary veins. *Right,* dilated LA. The left superior and left inferior pulmonary veins join to form a common trunk before draining into the left atrium. *Left,* an example of an accessory pulmonary vein on the right side. This smaller pulmonary vein had firing potential, which was ablated.**


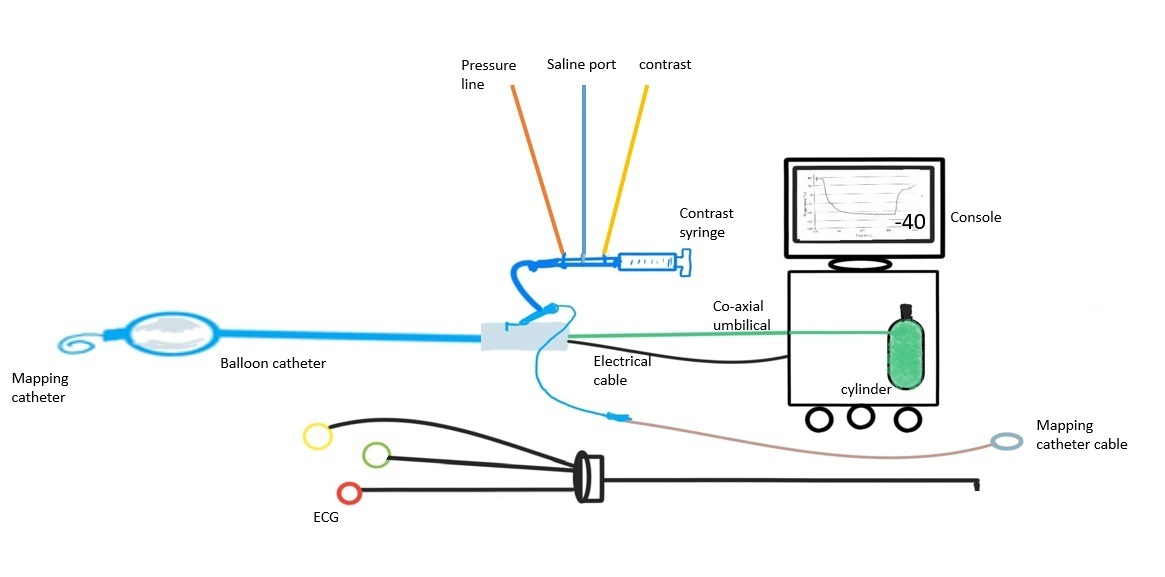


**Figure 10: Schematic drawing of cryoballoon ablation system, connections, and setup** *(not to scale)*
